# Intervention intended to improve public health professionals’ self-efficacy in their efforts to detect and manage perinatal depressive symptoms among Thai women: a mixed-methods study

**DOI:** 10.1186/s12913-020-5007-z

**Published:** 2020-02-24

**Authors:** Nitikorn Phoosuwan, Pranee C. Lundberg, Sadiporn Phuthomdee, Leif Eriksson

**Affiliations:** 1 Department of Public Health and Caring Sciences, Faculty of Medicine, Uppsala University, BMC, Husargatan 3, Box 564, 751 22 Uppsala, Sweden; 2 Department of Community Health, Faculty of Public Health, Kasetsart University Chalermphrakiat Sakonnakhon Province Campus, Sakonnakhon, Thailand; 3 Panyananthaphikkhu Chonprathan Medical Centre, Srinakarinwirot University, Nonthaburi, Thailand

**Keywords:** Depression screening tool, Health professional, Intervention programme, Self-efficacy

## Abstract

**Background:**

Targeting perinatal depressive symptoms among women can reduce premature mortality. However, public health professionals (PHPs) in primary healthcare settings often have low self-efficacy for detection and management of perinatal depressive symptoms among women. This mixed-methods study was aimed at developing and evaluating a self-efficacy improvement programme (SIP) intended to increase PHPs’ self-efficacy in efforts to detect and manage perinatal depressive symptoms.

**Methods:**

The SIP consisted of 1 day of theory and 4 weeks of practice. Sixty-six PHPs from sub-district health promotion hospitals (primary health care level) in Sakonnakhon, a north-eastern province in Thailand, were randomised into an intervention group (*n* = 33) and a control group (*n* = 33). Twenty-three of the intervention group participants also took part in focus group discussions (FGDs). Multiple linear regression and qualitative content analysis were used to analyse the data.

**Results:**

After the SIP, the intervention group participants had higher self-efficacy score than those in the control group (*p* = 0.004). The FGDs resulted in four categories emerging: Having confidence, Changing knowledge and attitudes, Increasing perception of an important role, and Increasing awareness of performed function.

**Conclusions:**

To enhance the ability of PHPs to detect and manage perinatal depressive symptoms, an intervention programme based on self-efficacy modification is recommended.

## Background

Worldwide, more than 10% of pregnant women and 21% of women after childbirth experience depressive symptoms [1, 2]. Improved mental health of women not only contributes to the third Sustainable Development Goal, reduction of premature mortality [3], but also to the strengthening of mother-child relationships and to the reduction of infant growth impairment [4]. Perinatal depressive symptoms can be alleviated by screening the population-at-risk and by correct management [5]. Currently, however, women remain under-screened and have not been well managed for depressive symptoms during the perinatal period (i.e. during pregnancy and after childbirth) in many maternity care settings [4–6]. Moreover, more than 80% of women with perinatal depressive symptoms do not seek professional assistance due to lack of knowledge [7].

In resource-constrained primary healthcare centres, detection and management of depressive symptoms by trained healthcare providers (HCPs) targeting women during the perinatal period can reduce the number of women with symptoms of depression [5, 8]. However, several barriers exist for this to take place, e.g. lack of knowledge about perinatal depressive symptoms, how to use screening tools such as the Edinburgh Postnatal Depression Scale (EPDS) and how to refer women for diagnosis and treatment [9, 10]. Intervention programmes about detection and management of perinatal depressive symptoms targeting HCPs can assist in overcoming these barriers and potentially have long-term impacts on mothers and children, e.g. maternal health and child development [6, 7].

In Thailand, primary healthcare centres are named sub-district health promotion hospitals (SHPHs). In an SHPH, there are about four HCPs, i.e. nurses/midwives with a bachelor’s degree in nursing and midwifery, public health professionals (PHPs) with a bachelor’s degree in public health, and public health assistants with a diploma degree in public health/dental health. HCPs at SHPHs provide health promotion, disease prevention, treatment and rehabilitation. A nurse/midwife mainly provides antenatal care (ANC). Currently, maternal depression screening is performed once during pregnancy by the nurse/midwife using a two-question tool (2Q) [11]. The 2Q has been developed by Thai experts for depression screening in the general Thai population [12]. However, in order to properly identify perinatal depressive symptoms among women, a specific screening tool should be implemented [13]. Screening for perinatal depressive symptoms is a preventive action, which could be a routine task for a PHP [8]. To achieve this, PHPs need to be trained and supervised in order to increase their self-efficacy when providing mental health promotion and screening [14–16].

The self-efficacy theory by Bandura [17] focuses on efficacy expectations to improve a person’s behaviour. The efficacy expectations may be increased by four information sources: performance accomplishment, vicarious experience, verbal persuasion and physiological states. Performance accomplishment is generated when a person has the correct knowledge and attitude; vicarious experience is experience from a person who is successful in a specific job; verbal persuasion comes from suggestions; and physiological states could come from non-verbal actions. The expectations can, for example, be measured by the Generalized Self-Efficacy Scale, which measures goal setting, effort investment, persistence in face of barriers, and recovery from setbacks [18]. Self-efficacy among HCPs is a key factor for successful detection of maternal depression in a community [9]. It can be improved by an appropriate intervention programme [19].

In order to improve self-efficacy among PHPs in detection and management of perinatal depressive symptoms, a self-efficacy improvement programme (SIP) was developed. This study was aimed at developing and evaluating an intervention programme intended to increase PHPs’ self-efficacy in efforts to detect and manage perinatal depressive symptoms.

## Methods

### Study design and setting

This mixed-methods study has quantitative (randomised controlled trial, RCT) and qualitative (explorative) components in order to get a more comprehensive understanding of the self-efficacy of PHPs after participating in an intervention programme [20]. The quantitative method will demonstrate changes of self-efficacy scores of PHPs in their efforts to detect and manage perinatal depressive symptoms during a self-efficacy improvement programme, while the qualitative method will assist in further exploring the process of implementing the intervention programme. The study was conducted in Sakonnakhon, a north-eastern province of Thailand. Sakhonnakhon has eighteen districts, with approximately 300 PHPs working in 168 SHPHs. Annually, there are approximately 13,000 childbirths in this province [21].

### Self-efficacy Improvement Programme (SIP)

The SIP developed by the researchers was based on Bandura’s self-efficacy theory [17, 22]. It contained two parts: (1) 1 day of theory and (2) 4 weeks of practice. See Table 1.

**Table 1:** Contents of the Self-efficacy Improvement Programme in relation to the four information sources of Bandura’s self-efficacy theory [17, 22]

Information source	Contents	Implementation
Performance accomplishment	Knowledge about associated factors and screening for perinatal depressive symptoms among women	One day of theory lectured by a public health expert, group work by the participants
	Outcomes of detection and management of perinatal depressive symptoms	One day of theory lectured by a public health expert
Vicarious experience	Experience sharing in the perinatal depressive symptom management: referral system, diagnosis, treatment options, community engagements	One day of theory lectured by a psychiatric nurse, panel discussion
	Screening and management for perinatal depressive symptoms among women in the community	Four weeks of practice by the participants
Verbal persuasion	Motivation for detection and management of perinatal depressive symptoms	One day of theory lectured by an expert in health education and behavioural science
	Supervision of the participants by phone and mobile applications	Four weeks of practice by a public health expert
Physiological states	Presentation of a manual for psychosocial management and guidelines for detection and management of perinatal depressive symptoms	One day of theory lectured by a public health expert
	The use of a questionnaire to screen perinatal depressive symptoms and its associated factors	One day of theory trained by a public health expert and practiced in pairs by the participants)
	Supervision of the participants by face-to-face visit	Four weeks of practice by a public health expert

The day of theory was arranged at the Sakhonnakhon Provincial Public Health Office. It included 8 hours of interactive lectures (devoted to the four information sources from the self-efficacy theory [17]), with focus on the knowledge of motivation for and outcome of detection and management of perinatal depressive symptoms. A manual for psychosocial management from the World Health Organisation (WHO) [23] translated by the first author (NP), with guidelines for detection and management of perinatal depressive symptoms constructed by the authors, and a questionnaire for perinatal depressive symptoms were introduced to the participants by three speakers with experience in psychiatric nursing, health education and behavioural science, as well as public health. The questionnaire comprised the Thai-validated EPDS screening tool for women during antenatal and postnatal periods [24–26], psychological well-being questions [27], self-esteem questions [28], and sense of coherence questions [29]. These tools were introduced according to our previous studies of risk factors for perinatal depressive symptoms among women in a north-eastern province of Thailand [24, 25]. The participants were also given material for practice, such as manuals and questionnaire copies.

The four weeks of practice started directly after the day of theory. During the practice, the participants of the intervention group were asked to practice with at least two women (one pregnant woman and one woman after childbirth) in their community/SHPH according to the manual and guidelines provided during the day of theory. In order to assist the participants, NP supervised each participant once by phone. In addition, all participants were supervised by NP by mobile applications (Facebook and Line), and field visits were arranged for those who needed face-to-face assistance.

### Participants

#### Quantitative considerations

The PHPs were selected randomly from SHPHs in six districts in Sakonnakhon [21]. To be eligible, PHPs should work in the selected districts and be willing to participate in the study. The sample size was determined for RCT with continuous variable [30] (power of test = 80%, effect size = 20%, a set of 95% significance level, standard deviation (SD) = 4.3, mean difference = 1.1 and superiority margi*n* = 4 based on the self-efficacy score among PHPs for detection and management of perinatal depressive symptoms from our pilot study in a province near Sakonnakhon). The required number of participants was at least 28 per group. To compensate for a possible 15% loss in the follow-up, it was decided to include 33 participants per group.

#### Qualitative considerations

After the 4 weeks of practice, 23 out of the 33 PHPs in the intervention group participated in four focus group discussions (FGDs): FGD1 (*n* = 4), FGD2 (*n* = 8), FGD3 (*n* = 6) and FGD4 (*n* = 5). Reasons for not participating in the FGDs were other urgent meetings, lack of time and the work situation at their SHPH.

### Procedure

Initially, the research proposal was sent to the Ethics Committee in Sakonnakhon for approval (SWDCPH2017–003). The head of Health Promotion Department of Sakhonnakhon Provincial Public Health Office approved the intervention, and the heads of the District Public Health Offices approved the data collection. Thereafter, invitations containing questionnaires and information about the study were distributed to PHPs by ordinary mail in the six selected districts in Sakonnakhon with high annual number of childbirth. The questionnaire was used to collect baseline data (T1), and the information explained details about the study and contained informed consent forms. Before the study, the PHPs were informed in writing about the purpose of the study, that their participation was voluntary, and that they could drop out from the study at any time. In total, 134 PHPs agreed to participate in the study. Out of them, 33 were randomly allocated to the intervention group and 33 to the control group. Sixty-eight PHPs had to be excluded for reasons such as inability to fully participate. The intervention group members participated in the SIP, while those of the control group worked as usual.

To evaluate the differences between the self-efficacy scores before and after the SIP, the participants of the intervention group were asked to complete a questionnaire on four occasions: during baseline (T1), immediately before the day of theory (T2), immediately after the day of theory (T3) and immediately before the FGDs (T4). The participants of the control group were asked to complete the questionnaire on two occasions: during baseline (T1) and close to the time of T4.

**Fig. 1:**
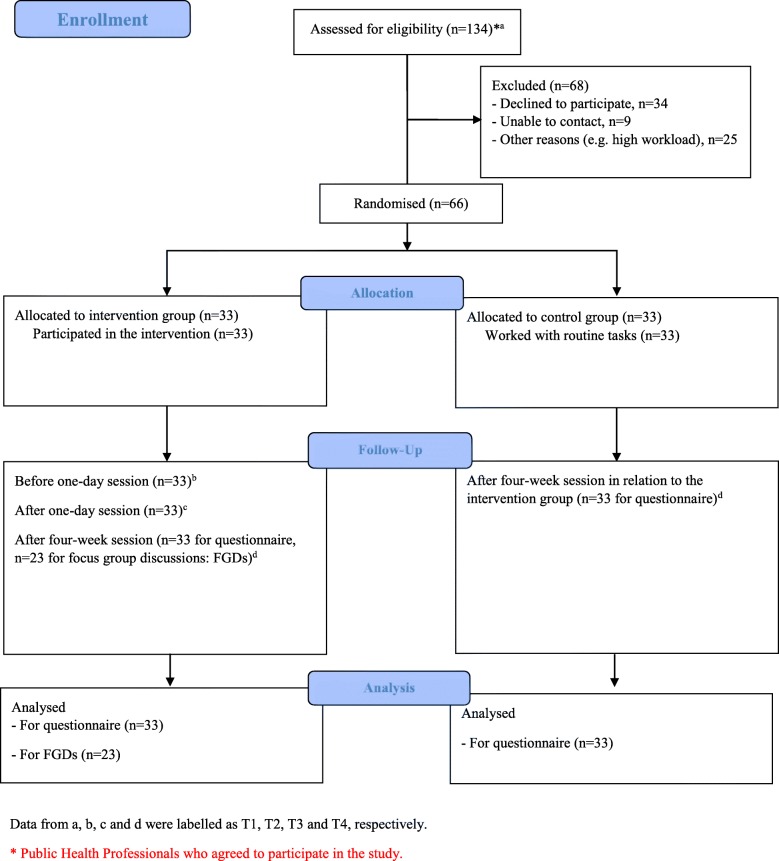
The flow diagram of the study

The FGDs were carried out at the provincial and district public health offices at a convenient date. Each FGD, with participation of the FGD members, NP and PCL, lasted about 2 hours. NP is male with a master degree in public health and experience in qualitative research, while PCL is female with a PhD degree and experience of nursing/midwifery and qualitative research. They had no relation with the participants before the study was conducted. NP acted as a moderator, while PCL took notes and asked additional questions. During the FGDs, the participants were asked to make clarifications when needed. The discussions were tape-recorded and then transcribed verbatim. All transcripts were given codes without name identification. A flow diagram of the study is shown in Fig. 1.

### Data collection

#### Collection of quantitative data

The questionnaire contained questions about sociodemographic characteristics and self-efficacy. The sociodemographic part was developed by NP. It comprised the variables age, gender, marital status, education level, experience of mental health training, years working at the SHPH and size of the SHPH. The self-efficacy part originated from the Thai-translated version [31] of the Generalized Self-Efficacy Scale [18]. In order to assess self-efficacy of PHPs in their efforts to detect and manage perinatal depressive symptoms, the content of the scale was adjusted according to recommendations to capture self-efficacy [32]. The questionnaire had ten items, each scoring from one to four. Thus, the total score ranged from 10 to 40, with higher score indicating higher self-efficacy. Three public health experts approved the content of the questionnaire (Content Validity Index: CVI > 0.80). Internal consistency was performed among 30 PHPs who worked in another province of Thailand, with a Cronbach’s alpha coefficient of 0.959.

#### Collection of qualitative data

In the FGDs, the participants of the intervention group described their self-efficacy after they had participated in the SIP. An interview guide constructed by the authors was used to guide the FGDs. It had been tested and adjusted in the first FGD, which was included in the qualitative analysis. The questions in the interview guide were open-ended, as shown in Table 2.

**Table 2:** Interview questions used in focus group discussions (FGDs)

1. What are your experiences of meeting women with antenatal depressive symptoms in your community/health centre? 2. Please share your experiences from participating in the one-day theory of the self-efficacy improvement programme (SIP). 3. Please share your experiences from participating in the four-week field practice of the SIP. 4. How has your knowledge, attitude and self-efficacy changed after you participated in the SIP? 5. For evaluation purposes, please give your opinion of the SIP. 6. Is there something we did not discuss that you would like to add to the programme?	

### Data analyses

#### Quantitative analysis

Descriptive statistical analysis was performed using, e.g., mean and SD. Baseline sociodemographic characteristics and self-efficacy scores were compared between the intervention and control groups using Pearson chi-square, and Fisher’s exact and independent sample t- tests.

The main exposure was the SIP, and the outcome was the self-efficacy score at T4. Linear regression assumptions were checked (i.e. linearity, normality, multicolinearity and homoscedascity). Age, gender, marital status and number of years of work at the SHPH were entered to a multivariable linear regression analysis in order to adjust for potential confounding factors influencing the self-efficacy score. Self-efficacy scores at different times were compared for participants of the intervention group and for participants of the control group by use of paired sample t-test. The level of significance was set at 0.05, and 95% confidence interval was used.

#### Qualitative analysis

The transcripts were checked by NP to ensure their quality, and they were corrected before the analysis began. An inductive qualitative content analysis was performed, including three phases: preparation, organisation and report [33]. In the preparation phase, NP and PCL selected sentences to prevent fragmentation of words. Manifest content was chosen to maintain results as close to the text as possible. The transcripts were read repeatedly to familiarise ourselves with the texts. NP and PCL, both Thais, read the transcripts separately. The organisation phase comprised two processes: open coding and creation of categories. Open coding implied that notes and headings were written in the margins of texts to describe aspects of contents while reading the transcripts. Thereafter, categories were generated from the written notes and headings, and they were grouped considering their similarities and belongings. NP and PCL discussed the categories until agreement was reached. An example of the transcripts was translated into English in order to allow the non-Thai author (LE), who is male with a PhD degree and experience of primary healthcare nursing, implementation and qualitative research, to be engaged in the process. Final categories were agreed upon and reported to the participants in the FGDs. Quotations were presented in the results section and annotated with an abbreviation (FGD1–4).

## Results

### Quantitative results

At baseline, self-efficacy scores and sociodemographic characteristics among the participants of the intervention and the control groups were similar, with exception for age, marital status and number of years of work. See Table 3.

**Table 3:** Socio-demographic characteristics of participants at baseline

Characteristics	Intervention group (*n* = 33)	Control group (*n* = 33)	*p*-value
Age (year)			
Mean (SD)	32.21 (7.60)	40.12 (10.26)	<.01*^a^
Gender, n (%)			.08^b^
Male	11 (33.3)	18 (54.5)	
Female	22 (66.7)	15 (45.5)	
Marital status, n (%)			.013^b^
Single/widowed	20 (60.6)	10 (30.3)	
Married	13 (39.4)	23 (69.7)	
Training for mental health, n (%)			0.99^b^
Yes	3 (9.1)	4 (12.1)	
No	30 (90.9)	29 (87.9)	
Size of the Sub-district Health Promotion Hospital, n (%)			.05^c^
Small/medium	26 (78.8)	32 (97.0)	
Large	7 (21.2)	1 (3.0)	
Number of years of work			
Mean (SD)	7.19 (5.38)	11.52 (7.03)	.007*^a^
Self-efficacy score at baseline			
Mean (SD)	27.36 (3.32)	28.52 (5.46)	0.30^a^

^a^obtained by T-test; ^b^obtained by Chi-square test; ^c^obtained by Fisher’s exact test; *SD* Standard deviation; * statistically significant at 0.05 level

Linear regression analyses (crude and adjusted) were performed. The adjusted analysis demonstrated that after the SIP, the participants of the intervention group had higher self-efficacy score than those in the control group (β = 3.26, 95% CI 1.10–5.42, *p* = .004). See Table 4.

**Table 4:** Linear regression model for comparison of self-efficacy scores between intervention and control groups after four-week session (*n* = 66)

	Crude analysis	*P*-value	Adjusted analysis	*P*-value
	coefficient B (95% CI)		coefficient B (95% CI)	
Intervention	2.70 (0.78, 4.62)	.007*	3.26 (1.10, 5.42)	.004*
Age	–	–	0.04 (−0.13, 0.20)	.647
Marital status	–	–	−1.15 (−3.78, 1.48)	.385
Gender	–	–	−0.59 (−2.83, 1.64)	.598
Working year	–	–	0.42 (−2.68, 3.52)	.788

*SD* Standard deviation, *CI* Confidence interval

* statistically significant at 0.05 level

**Fig. 2:**
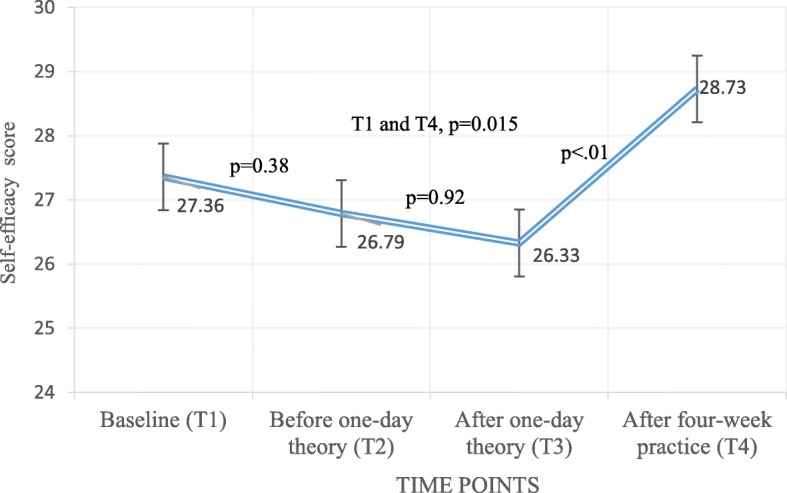
Changes in the self-efficacy scores among participants in the intervention group at baseline (T1), before one-day theory session (T2), after one-day theory session (T3) and after four-week practice session (T4)

For the participants of the intervention group, there were significant increases of the scores from T3 to T4, and from T1 to T4. However, no significant differences of the self-efficacy scores were detected from T1 to T2 or from T2 to T3 (Fig. 2). The self-efficacy score of the participants of the control group decreased from T1 to T4 (*p* = 0.002).

### Qualitative results

Overall, the participants in FGDs had positive experience from the SIP. The analysis resulted in four categories: Having confidence, Changing knowledge and attitude, Increasing perception of an important role, and Increasing awareness of performed function.

#### Having confidence

The participants in FGDs described that PHPs mainly work with communicable disease prevention and control, such as dengue control and surveillance. They disclosed a lack of confidence to work with depressive symptoms, particularly among women during the perinatal period. After the SIP, the participants expressed that having guidelines for screening and management of perinatal depressive symptoms from the intervention programme gave them confidence because they explained theories and how to work in practice. The participants felt that the guidelines were clear. Therefore, they felt it was easy to give women advice and screen for perinatal depressive symptoms. Participants also felt that they were able to explain how to prevent perinatal depressive symptoms when working together with their colleagues (i.e. nurses and other PHPs). They further mentioned that the face-to-face supervision during the 4 weeks of practice was important and increased their confidence.

*I gave the guidelines and screening questionnaire to my colleague [a nurse] who is responsible for pregnant women and explained to her how to use the guidelines and how to promote mental health for pregnant women.* (FGD1)

*The guidelines have clear and appropriate contents for us [PHPs]. I read the guidelines before I worked with women (during pregnancy and after childbirth). I am sure that I can advise women if they are at risk of getting depressed… I think that the supervision has been alright, but it should be longer.* (FGD4)

#### Changing knowledge and attitude

The participants had limited knowledge about perinatal depressive symptoms before they participated in the SIP. They mentioned that the interactive lectures during the day when the theory was covered provided new knowledge about perinatal depressive symptoms, e.g. how to use standardised tools, and how to prevent such symptoms.

*We [PHPs] at SHPHs screened for depressive symptoms using a two-question depression screening tool for the general population because we only had basic knowledge … Previously, I thought that depressive symptoms occurred among pregnant women but that the symptoms would disappear after childbirth. Now, after the training, I know that depressive symptoms can occur among women, both during pregnancy and after childbirth.* (FGD2)

Most of the participants described that they used to focus on the pregnant women’s physical condition at the SHPHs, e.g. to perform a physical examination and suggest treatments. However, due to inspiration from the day of theory, perinatal depressive symptoms now get more attention.

*Before the SIP, I focused only on the pregnant women’s physical health, not on their mental health. But after the intervention programme, I provide more mental health promotion for the women.* (FGD1)

#### Increasing perception of an important role

The participants described that PHPs usually worked with mental health tasks guided by government and local policies. However, most PHPs at the SHPHs did not know who should be responsible for preventing perinatal depressive symptoms. For instance, when pregnant women came to the SHPHs, some PHPs assisted the nurse/midwife to count the number of ANC visits, while other PHPs checked pregnant women’s vital signs. After the SIP, the PHPs understood that their most important task is to screen and manage perinatal depressive symptoms, because women in the communities know and trust them. They experienced that the SIP had benefits and should be offered to all PHPs in the SHPHs. In this way, every PHP could have the same level of knowledge, which also would reduce the workload of other professionals.

*I think that the intervention programme should have more than one day of theory… The programme should be disseminated to PHPs in other SHPHs because all PHPs should know about perinatal depressive symptoms and look for mental health problems among pregnant women. Also, if one PHP is absent, the other PHPs can advise the pregnant woman about mental health instead of just letting her go back home.* (FGD1)

#### Increasing awareness of performed function

Most of the participants described that providing mental health services during ANC had been perceived as complicated and difficult due to lack of mental health specialists, tools and procedures. They mentioned that the current screening tool for depression (2Q) could not detect perinatal depressive symptom cases properly. After the SIP, the participants planned to adapt their tasks by using the internationalised depression screening questionnaire from the SIP as a tool for screening women, both during pregnancy and after childbirth. They also planned to include women with depressive symptoms into the home visit plan for the multidisciplinary team at the SHPHs in order for pregnant women to receive both physical and mental health services.

*I suppose that we will use this developed screening questionnaire. It could increase the effectiveness of our work. Also, if screening would be used in all trimesters of pregnancy, I think we could get a better idea of the mental health situation in the community.* (FGD1)

*If we knew that this woman is at risk during pregnancy or after childbirth, we would plan with the multidisciplinary team to follow-up and provide more specific support to her.* (FGD3)

## Discussion

Previous studies indicate that HCPs in government settings have insufficient self-efficacy to provide care in the communities and are in need of more training [14–16]. Self-efficacy can improve work performance [16]. The self-efficacy theory is widely applied to facilitate behavioural modifications [14] and an appropriate intervention programme has the possibility to increase self-efficacy among PHPs in local communities [16]. The present study revealed that self-efficacy of PHPs in their efforts to detect and manage perinatal depressive symptoms was significantly improved after participation in the SIP, an intervention programme containing both theoretical and practical parts. In the SIP, all four information sources from the self-efficacy theory by Bandura [17] were emphasised and the psychosocial management manual from WHO [23] was provided. One day of theory gave the PHPs in the intervention group opportunity to share their experiences with persons who had been successful in perinatal depressive symptom management, while the 4 weeks of practice gave the PHPs vicarious experiences in screening and management of perinatal depressive symptoms, e.g. giving advice to women who were at risk in the community. The participants’ self-efficacy for detection and management of depressive symptoms among women increased, which is an important factor to improve the situation for women during the perinatal period [8, 16, 24]. The results demonstrate that the four information sources from the theory by Bandura [17] are key components for improvement of self-efficacy among PHPs.

The self-efficacy scores among the participants in the intervention group were unchanged between baseline (T1) and directly after the theoretical training (T3), but they were significantly increased between T3 and T4 (after the four-week practice). To increase self-efficacy, it seemed necessary for PHPs to practice what they had learned in theory. This is related to the performance accomplishment in Bandura’s theory [17]. Previously, it has also been shown that HCPs with experience from patient practice increase their motivation to work on preventive tasks with women [34]. The FGDs revealed that the participants gained confidence in how to detect perinatal depressive symptoms because they had practiced with at least two women. The participants also had confidence in how to manage perinatal depressive symptoms, i.e. to advise women with an EPDS score ≥ 10 to meet a physician for diagnosis and treatment. This is in line with previous studies showing that training HCPs on how to detect and manage perinatal depressive symptoms can increase HCPs’ confidence when working in primary healthcare centres [9, 10, 16]. The participants also highlighted the importance of the supervision visits during the SIP. These visits in the SIP might be viewed as an information source in the self-efficacy theory of Bandura [17]. Others also expressed that site visits are essential in order to promote better behaviour during an intervention programme [35]. Follow-up sessions can improve confidence to provide better care [36] and encourage self-efficacy to manage behaviour in a better way [37]. Individuals who are well informed and motivated are likely to achieve better outcomes [38]. Hence, based on this study and other research, it seems to be important to integrate supervision in the field practice into a self-efficacy modification intervention programme.

PHPs in the intervention group described that their knowledge and attitude had been increased by their participation in the SIP. Knowledge is a source of personal competence and attitude is a belief to provide a specific service [39]. Correct knowledge and attitudes among HCPs are key factors for perinatal mental health actions [9]. Lack of knowledge among HCPs is related to lack of self-efficacy [40], and negative attitude among HCPs may compromise the motivation of depression prevention [39]. Knowledge and attitudes are information sources in the self-efficacy theory [22], and they were both targeted in the SIP. Many intervention programmes focus on improving either knowledge or attitude of HCPs [39, 40]. The current study provides evidence that both knowledge and attitude changed positively by the SIP.

After completion of the SIP, participants in the intervention group perceived that they should screen women for depressive symptoms during the perinatal period using the EPDS because detection and management of depressive symptoms were their responsibilities. This finding is in line with a previous study, suggesting that community health workers are suitable persons to detect and manage perinatal mental health among women [8]. However, the FGD participants also described that HCPs already have a high workload as few HCPs appear in SHPHs, and mental health tasks have not been on their agenda. This situation is not unique for the province of the current study; it has been reported from many settings, particularly in middle-income countries [12, 15]. Nevertheless, universal screening needs to be implemented nationwide and be accessible for all women during the perinatal period [5, 7, 13]. After participation in the SIP, PHPs became more aware of their function at the SHPHs and intended to integrate perinatal mental health tasks into their daily practice. This implied involving women with depressive symptoms in their home visit plan, and increasing accessibility and continuity of care for women with mental health problems in the community [6, 9]. Management of women with depression symptoms at a primary care level, should include referral of women with high EPDS scores (≥ 10) for treatment and diagnosis by physicians, which may result in decreased depression severity [8, 24].

### Strengths and limitation

A strength of this study was the use of both quantitative and qualitative methods to evaluate the self-efficacy intervention programme [20]. Many factors influenced the perception of self-efficacy among HCPs, including marital status [41], number of years of work and age [16]. These are potential confounders, which have been controlled for in the analysis. This study was a RCT, which help to manage for biases [42]. In addition, by using the qualitative method, the experiences among participants in the intervention programme were explored more in-depth, such as their confidence and application. Trustworthiness was enhanced by having discussions of the results with Thai and non-Thai researchers, by conducting the study with researchers having experiences in public health and nursing-midwifery, by having an interview guide with questions guiding the FGDs, and by having description of contents and methods.

Despite its strengths, the SIP was conducted in a short time period and only with a number of PHPs in a north-eastern province of Thailand. This aspect needs to be taken into consideration, e.g. follow-up PHP using screening tool and manual, and field practice during a period longer than 4 weeks. This might result in better improvement of PHPs’ self-efficacy scores for detection and management of perinatal depressive symptoms. A more thorough evaluation process [43] might have provided more input on the implementation of the SIP. The circumstance that PHPs in the intervention and control groups did not work at the same SHPHs reduced the risk for contamination between the groups. However, contamination could still happen as PHPs in the intervention and control groups worked in the same districts [44].

## Conclusions

The results of the study indicate that the SIP significantly improved the self-efficacy of PHPs in primary healthcare centres when attempting to detect and manage perinatal depressive symptoms. After participating in the SIP, PHPs in the intervention group reported positive changes in their confidence, knowledge and attitude. The SIP also increased their perception of having an important role and their awareness of performed function at their SHPHs. The intervention programme, supported by supervision, was able to increase the self-efficacy of PHPs in their efforts to detect and manage perinatal depressive symptoms among women. To enhance detection and management of perinatal depressive symptoms by PHPs in primary healthcare centres, a training programme based on self-efficacy modification is recommended.

## Data Availability

The datasets used and/or analysed during the current study are available from the corresponding author on reasonable request.

## Abbreviations

ANC: Antenatal Care
CVI: Content Validity Index
EPDS: Edinburgh Postnatal Depression Scale
FGD: Focus Group Discussion
HCP: Healthcare Provider
PHP: Public Health Professional
RCT: Randomised Controlled Trial
SHPH: Sub-district Health Promotion Hospital
SIP: Self-efficacy Improvement Programme
WHO: World Health Organisation
